# TIN2 Functions with TPP1/POT1 To Stimulate Telomerase Processivity

**DOI:** 10.1128/MCB.00593-18

**Published:** 2019-10-11

**Authors:** Alexandra M. Pike, Margaret A. Strong, John Paul T. Ouyang, Carol W. Greider

**Affiliations:** a Department of Molecular Biology and Genetics, Johns Hopkins University School of Medicine, Baltimore, Maryland, USA; b Graduate Program in Cellular and Molecular Medicine, Johns Hopkins University School of Medicine, Baltimore, Maryland, USA; c Graduate Program in Biochemistry Cell and Molecular Biology, Johns Hopkins University School of Medicine, Baltimore, Maryland, USA

**Keywords:** POT1, TIN2, TPP1, alternative splicing, processivity, shelterin, telomerase, telomere

## Abstract

TIN2 is an important regulator of telomere length, and mutations in *TINF2*, the gene encoding TIN2, cause short-telomere syndromes. While the genetics underscore the importance of TIN2, the mechanism through which TIN2 regulates telomere length remains unclear. Here, we tested the effects of human TIN2 on telomerase activity. We identified a new isoform in human cells, TIN2M, that is expressed at levels similar to those of previously studied TIN2 isoforms. All three TIN2 isoforms localized to and maintained telomere integrity *in vivo*, and localization was not disrupted by telomere syndrome mutations. Using direct telomerase activity assays, we discovered that TIN2 stimulated telomerase processivity *in vitro*. All of the TIN2 isoforms stimulated telomerase to similar extents. Mutations in the TPP1 TEL patch abrogated this stimulation, suggesting that TIN2 functions with TPP1/POT1 to stimulate telomerase processivity. We conclude from our data and previously published work that TIN2/TPP1/POT1 is a functional shelterin subcomplex.

## Abstract (precis)

TIN2 is an important regulator of telomere length, and mutations in *TINF2*, the gene encoding TIN2, cause short-telomere syndromes. While the genetics underscore the importance of TIN2, the mechanism through which TIN2 regulates telomere length remains unclear. Here, we tested the effects of human TIN2 on telomerase activity. We identified a new isoform in human cells, TIN2M, that is expressed at levels similar to those of previously studied TIN2 isoforms.

## INTRODUCTION

Telomere length in human cells is maintained around a tight equilibrium that prevents life-threatening disease. Telomere shortening leads to a characteristic set of degenerative diseases, including pulmonary fibrosis, bone marrow failure, and immune deficiency, collectively called short-telomere syndromes (1). In contrast, 90% of human cancers upregulate telomerase, and mutations that increase telomerase levels predispose to cancer (2–4). While we understand many component pathways that regulate telomere length, the integrated mechanism of telomere length regulation is not fully understood.

Human telomeres consist of about 10 kb of TTAGGG repeats that are mostly double-stranded DNA with a single-stranded 3′ overhang, all bound by a protein complex termed shelterin (5). This DNA-protein complex maintains telomere integrity, and shelterin both positively and negatively regulates telomere repeat addition by telomerase. The shelterin complex consists of the following six subunits: two double-stranded DNA binding proteins, TRF1 and TRF2 (6–9); a single-stranded telomeric binding protein, POT1 (10, 11), and interacting proteins TPP1, TIN2, and RAP1 (12–16).

POT1 and TPP1 form a heterodimer that binds single-stranded telomeric DNA and stimulates telomerase processivity *in vivo* and *in vitro* (17–19). This stimulation is mediated though the TPP1 OB-fold, which contains the conserved TEL patch and NOB regions that directly interact with the TEN domain of telomerase reverse transcriptase (TERT) (20–23). Mutations in the TEL patch abrogate the stimulation of processivity, and compensatory charge swap mutations in TERT restore function (24), suggesting the direct binding of TPP1/POT1 heterodimer to TERT mediates telomerase processivity stimulation.

**FIG 1:**
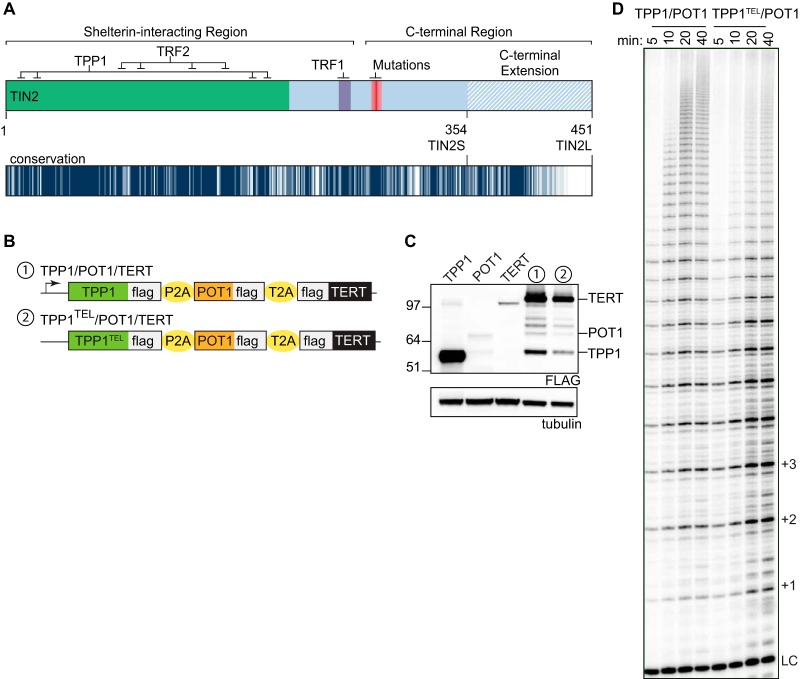
Generation of cell lines for extract-based *in vitro* telomerase assays. (A) Detailed schematic of the TIN2 protein. The TRF2/TPP1 interaction domain is indicated in green, with simplified TPP1 and TRF2 contacts illustrated at the top. A TRF1 FXLXP interaction motif is indicated in purple. The red gradient indicates the patient mutation cluster, where mutated residues cluster but differ in their frequency and disease severity. The blue hatched region indicates the variable C-terminal extension. At the bottom is a conservation track generated from the values from a multiple-sequence alignment performed with 35 known or predicted TIN2 proteins (see Materials and Methods and Table S1 in the supplemental material), with degrees of conservations indicated on a color scale ranging from white (score of 0 [not conserved]) to navy (score of 10 [highly conserved]). (B) Expression cassettes used in this study. All cassettes were expressed under the control of the cytomegalovirus (CMV) promoter in the pcDNA5/FRT backbone. Telomerase assay cell lines were generated as described in Materials and Methods. (C) Western blot of individually transfected TPP1, POT1, and TERT cDNAs next to telomerase assay cell lines with numbers corresponding to those in panel B. FLAG bands above POT1 are unidentified but may represent TERT degradation products. (D) Telomerase assays were stopped at 5, 10, 20, and 40 min for each cell line. Telomere repeats are indicated by +1, +2, etc. LC, loading and purification control.

TIN2, encoded by the *TINF2* gene, is essential for shelterin assembly at the telomere and is mutated in patients with severe short-telomere syndromes (25, 26). The biochemical function of or mechanism by which TIN2 regulates telomere length is not yet clear. TIN2 localizes to telomeres through interactions with TRF1, TRF2, and TPP1 (Fig. 1A) (12–15, 27). TIN2 interaction with TPP1 is essential for TPP1/POT1 localization and function in cells (28–31), and TIN2 stabilizes TRF1 and TRF2 binding to telomeres (27). Because of its interactions with TRF1, TRF2, and TPP1/POT1, TIN2 has been described as a molecular bridge between the double-stranded and single-stranded DNA-binding shelterin components. However, it is likely that TIN2 performs additional telomeric functions, since single missense mutations significantly disrupt telomere length equilibrium.

While mouse cells express a single TIN2 isoform, human cells express short and long alternatively spliced isoforms, TIN2S and TIN2L (Fig. 1A). Both isoforms contain the shelterin interacting domains that bind TRF1, TRF2, and TPP1, as well as the small domain of unknown function that is mutated in patients with short-telomere syndromes. TIN2L contains the entire TIN2S sequence (residues 1 to 354), along with a C-terminal extension that contains highly conserved residues (Fig. 1A). Recent evidence suggests that the longer isoform is functionally different from TIN2S (32), but TIN2S is the most widely studied isoform.

To elucidate the mechanism of TIN2 regulation of telomere length in human cells, we set out to test the biochemical functions of the TIN2 isoforms in the context of the TPP1/POT1 telomerase processivity complex. We found that TIN2 stimulates telomerase processivity in a TPP1/POT1-dependent manner. Further, we found a third isoform, TIN2M, expressed in human cells. Each TIN2 isoform can localize to telomeres, maintain telomere integrity, and stimulate telomerase processivity. Together with the requirement of TIN2 for TPP1/POT1 function *in vivo*, these data implicate TIN2 as a part of the telomerase processivity complex, forming a functional shelterin subcomplex. This is the first evidence of TIN2 having a direct role in regulating telomerase, suggesting that it may do more than simply bridge the shelterin components at the telomere.

## RESULTS

To understand the mechanism of TIN2 in telomere length regulation, we examined whether TIN2 might directly affect telomerase activity. TIN2 interacts directly with the TPP1 component of the TPP1/POT1 telomerase processivity complex that binds telomerase through the TPP1 TEL patch domain (18, 20–22, 33). To examine whether TIN2 affects telomerase activity or processivity, we reconstituted active telomerase complexes in a cell extract-based system overexpressing telomerase to measure telomerase activity and processivity *in vitro* (18–20, 34). We adapted this previously published extract-based system (20) to generate cells constitutively expressing telomerase (TERT/TR), TPP1, and POT1 and then introduced TIN2 by transient transfection.

For reproducible overexpression of the protein components, we created a polycistronic expression cassette containing FLAG-TPP1, FLAG-POT1, and FLAG-TERT separated by 2A peptides (Fig. 1B). As a negative control, we mutated the TPP1 TEL patch (TPP1 E169A/E171A) (20), referred to here as TPP1^TEL^, to test whether any effects of TIN2 are mediated through TPP1/POT1 (Fig. 1B). We constructed the assay cell lines by first generating a clonal cell line overexpressing telomerase RNA (TR) in 293TREx FLP-in cells, into which we integrated the expression cassettes at a unique genomic locus using the FLP-in system. The resulting cell lines are referred to here as TPP1/POT1/TERT and TPP1^TEL^/POT1/TERT (Fig. 1B to D). Exogenous TIN2 was introduced to these cell lines by transient transfection (see Materials and Methods). We note that in this assay, relative to the exogenous proteins, levels of endogenous shelterin proteins are too low to affect the biochemical assays, as evidenced by the observation that endogenous TPP1 is unable to compensate for the processivity defects of TPP1^TEL^ in the TPP1^TEL^/POT1/TERT cell line (Fig. 1D).

**FIG 2:**
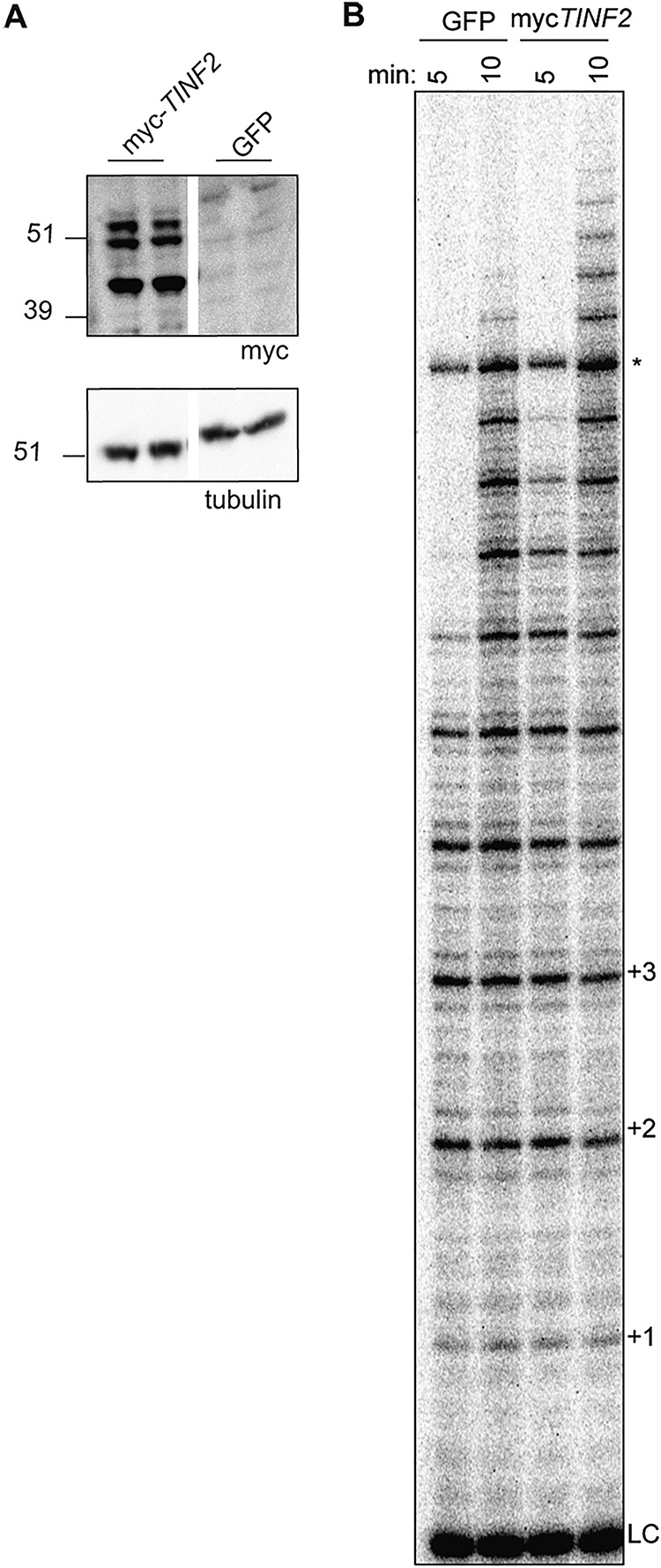
*TINF2* expression stimulates telomerase processivity. (A) Western blot of duplicate transfections of myc*TINF2* or GFP into TPP1/POT1/TERT cell lines. “*TINF2*” refers to the full-length gene, inclusive of introns. Samples were run on the same blot; the white line indicates cropping of lanes with samples not included in this study. (B) Telomerase assays of transfections performed as described for panel A were stopped after 5 or 10 min. LC, 18-mer loading and purification control; +1, +2, etc., repeat numbers; *, nonspecific band.

To examine the effects of TIN2 on telomerase activity, we initially transfected a myc-tagged full-length *TINF2* gene, inclusive of introns, to express both alternatively spliced TIN2 isoforms. Telomerase assays showed increased processivity in TPP1/POT1/TERT cells transfected with myc-*TINF2* compared to the green fluorescent protein (GFP) control (Fig. 2B), suggesting that TIN2 enhances telomerase processivity to a level above that resulting from effects of TPP1/POT1 alone. Surprisingly, Western blotting performed with these lysates showed three distinct bands, instead of the expected two bands corresponding to TIN2S and TIN2L (Fig. 2A). We deduced that the third band may correspond to a new isoform and, further, that any of the three isoforms could be responsible for the increased processivity in myc-*TINF2* lysates.

### Identification of a new TIN2 isoform, TIN2M.

**FIG 3:**
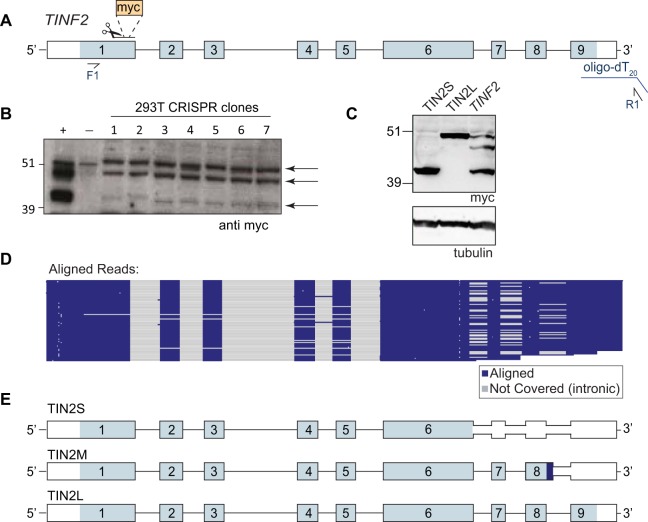
TIN2 has three predominant isoforms in human cells. (A) Schematic of CRISPR and PacBio experiments. Scissors indicate the location of cut site and myc tag insertion. Blue line, oligo(dT_20_)-adapter used for reverse transcription; F1 and R1, primers used for PacBio sequencing (reported in panels D and E). (B) Western blot of edited myc-TIN2 CRISPR clones 1 to 7. +, positive-control transfection of myc-*TINF2* plasmid; −, parental cell lines. Arrows indicate three distinct bands from TIN2 isoforms. Endogenous myc is apparent in the negative-control lane and runs at a size similar to that of TIN2L. (C) Myc Western blot of overexpressed cDNA for TIN2S and TIN2L and the full-length myc-*TINF2* gene. Tubulin is shown as a loading control. (D) PacBio sequencing track showing the coverage of TIN2 exons followed by aligned sequence reads shown in blue and gray. Each line represents a single read. Blue; aligned sequence; gray, not covered (introns and indels). (E) StringTie-generated TIN2 transcripts from combined data from 293T, HeLa, RPE-1, K562, and LCL cell lines showing TIN2S, TIN2L, and the new isoform, TIN2M. Light blue, coding sequence; dark blue, unique TIN2M sequence; white, untranslated region.

To further examine the TIN2 isoforms, we engineered an N-terminal myc tag at the endogenous *TINF2* locus in 293T cells using CRISPR/Cas9 genome editing (Fig. 3A). Western blots on several edited clones again showed the three distinct bands (Fig. 3B), suggesting that the third band did not represent an artifact of the overexpression construct. To identify these bands, we transfected myc-*TINF2* alongside cDNA constructs expressing myc-TIN2S or myc-TIN2L (Fig. 3C), which revealed that the unknown band fell between the known isoforms, running at ∼47 kDa (Fig. 3C).

To test whether this band corresponds to an alternatively spliced TIN2 isoform, we used a modified 3′ rapid amplification of cDNA ends (3′RACE) assay with PacBio sequencing to identify all full-length transcripts in human and mouse cells. In 293T cells, TIN2S and TIN2L cDNAs were identified along with a third major isoform, which encodes a protein with the expected molecular weight for the unknown protein. We termed this isoform “TIN2M” for “TIN2 medium.” TIN2M results from retention of the last intron, between exons 8 and 9, which encodes 13 amino acids of unique sequence (_408_VSGKEQKAGKGDG_420_) before reaching a stop codon (Fig. 3D and E). TIN2M was present at similar levels in four other human cell lines (HeLa, K562, RPE-1, and a newly derived lymphoblastoid cell line [LCL]), suggesting that it is commonly expressed.

Sequence read counts indicated that TIN2M and TIN2L mRNAs were expressed at similar levels, while TIN2S had 2-fold-greater representation than each of the others (Fig. 3D). These ratios are not always reflected in Western blots (Fig. 3B and C) as the mRNA abundance and protein levels may be discordant due to posttranslational regulation. Additionally, TIN2L has shown poor solubility and its detection varies depending on the protein extraction method (35).

In addition to the three major isoforms in human cells, we identified a number of additional recurrent exon skipping, intron retention, and alternative polyadenylation site usage events, including exon 2 skipping, described previously (36) (Fig. 3D). We found that two different mouse strains (C57BL/6 and CAST/EiJ) expressed just one TIN2 isoform that is most similar to TIN2L, as previously described (35, 37) (not shown).

Evidence for expression of TIN2M was also found in data publicly available from PacBio IsoSeq from MCF-7 breast cancer cells (http://www.pacb.com/blog/data-release-human-mcf-7-transcriptome/). Additionally, genome-wide ribosome profiling data from GWIPS-viz showed ribosome peaks present in the unique coding region of the TIN2M-retained intron (38). TIN2M and TIN2L contain the recently identified CK2 phosphorylation site (32), but TIN2S does not. All three of the expressed isoforms contain the documented cluster of telomere syndrome patient mutations and the other known interaction domains.

### All three TIN2 isoforms localize to telomeres.

**FIG 4:**
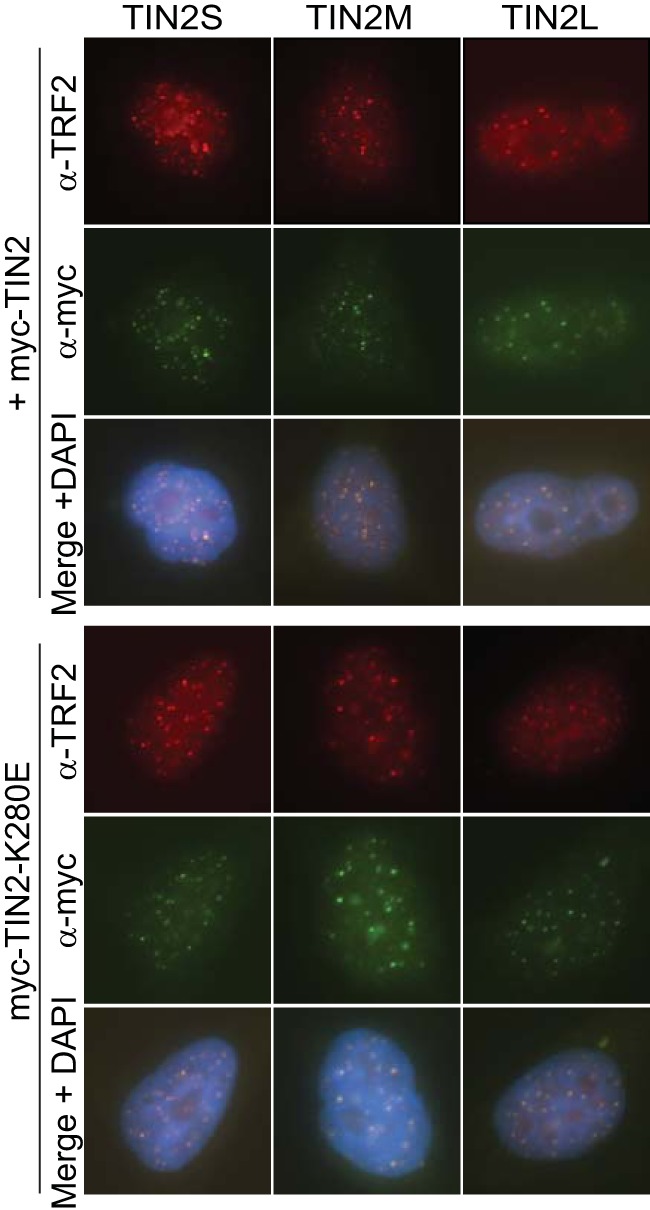
All TIN2 isoforms localized to telomeres. Images present immunofluorescence of cell lines expressing individual TIN2 isoforms. TRF2 marks telomeres (red), anti-myc antibody marks TIN2 (green), and nuclei were counterstained with DAPI. Merged images show telomeric foci with colocalized TRF2 and TIN2 staining for all three isoforms.

TIN2S and TIN2L have been demonstrated to localize to telomeres *in vivo* (12, 35). To determine whether TIN2M also localizes to telomeres, we stably expressed cDNA encoding myc-tagged TIN2S, TIN2M, or TIN2L in HeLa Flp-in cells. Using indirect immunofluorescence, we found that all three isoforms showed discrete foci that colocalized with TRF2, indicating that each localizes to telomeres *in vivo* (Fig. 4). This demonstrates that all three TIN2 isoforms may be found at endogenous telomeres in human cells and that the different C-terminal domains do not appear to affect telomeric localization.

Since all of the TIN2 isoforms also contain the patient mutation cluster (Fig. 1A), we tested whether mutant TIN2 is defective in telomeric localization. TIN2S with patient mutations K280E, R282S, and R282H was previously shown to localize to telomeres (39, 40), but localization in TIN2M or TIN2L with mutations has not been reported. We expressed a well-studied patient mutation, K280E, in each of the three isoforms and observed telomeric localization of all three isoforms with this mutation (Fig. 4). The results suggested that any of these three isoforms could mediate the dominant-negative telomere-shortening effect of the patient mutations *in vivo*.

### TIN2M and TIN2L rescue telomere damage foci.

**FIG 5:**
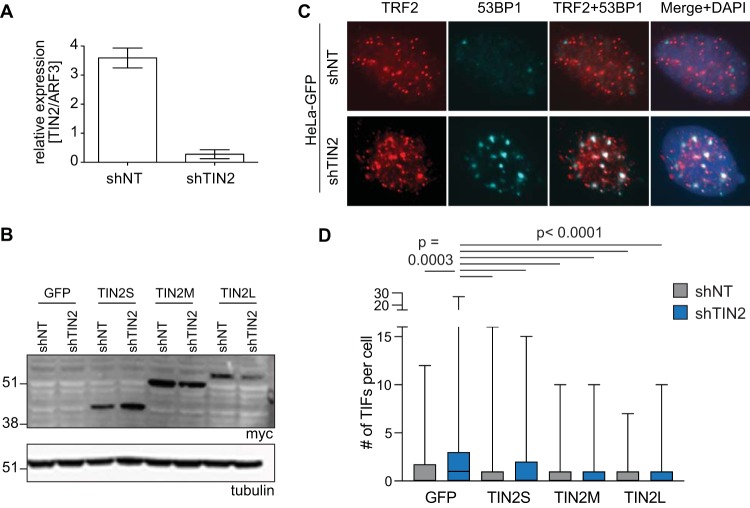
All three TIN2 isoforms rescued TIF formation caused by TIN2 knockdown. (A) qRT-PCR was performed on HeLa GFP-expressing cell lines transduced with nontargeting (shNT) or TIN2-targeting (shTIN2) shRNA lentiviruses. Expression level was analyzed by 2^−ΔΔ^*^CT^* relative quantification. shNT, *n* = 2; shTIN2, *n* = 3. (B) Western blot of HeLa cell lines expressing GFP or individual mycTIN2 isoforms, transduced with shNT or shTIN2 lentivirus. (C) Representative images of HeLa GFP cell lines transduced with shNT or shTIN2 lentivirus as indicated. Red, TRF2; cyan, 53BP1. TIFs are defined by colocalization of TRF2 and 53BP1. (D) Quantification of TRF2/53BP1 colocalizations per nucleus (TIFs), represented as a box plot with whiskers inclusive of all values (*n* > 250 nuclei per cell line). Data were analyzed with a Kruskal-Wallis one-way analysis of variance with Dunn’s correction for multiple comparisons. Significant *P* values are indicated on the graph.

TIN2 is an integral part of the shelterin complex, and disruption of shelterin results in a DNA damage response at telomeres, termed telomere dysfunction-induced foci (TIFs). Removal of endogenous TIN2 elicits a TIF phenotype that can be rescued by reintroducing TIN2 (28, 31). To determine whether each isoform can rescue this phenotype, we depleted endogenous TIN2 with short hairpin RNA (shRNA) in the HeLa myc-TIN2 cell lines described above using HeLa GFP as a control. The TIN2 shRNA (shTIN2) resulted in ∼90% knockdown of endogenous TIN2 compared to a nontargeting shRNA (shNT) in the GFP cell line (Fig. 5A) and did not target exogenous myc-TIN2 isoforms as detected by Western blotting (Fig. 5B).

In control cell lines, TIN2 knockdown significantly increased levels of TIFs compared to the nontargeting control results, as measured by colocalization between TRF2 and 53BP1 (Fig. 5C and D). This was reflected by a higher mean number of TIFs per nucleus as well as by a higher proportion of cells having a large number of TIFs. Overexpression of TIN2S prevented this increase in TIFs, and we found that TIN2M and TIN2L also effectively rescued TIFs when endogenous TIN2 was knocked down (Fig. 5D). This suggests that the three isoforms are individually capable of performing the telomere maintenance roles seen with TIN2 (Fig. 5C and D).

### TIN2 cooperates with TPP1/POT1 to stimulate telomerase processivity.

**FIG 6:**
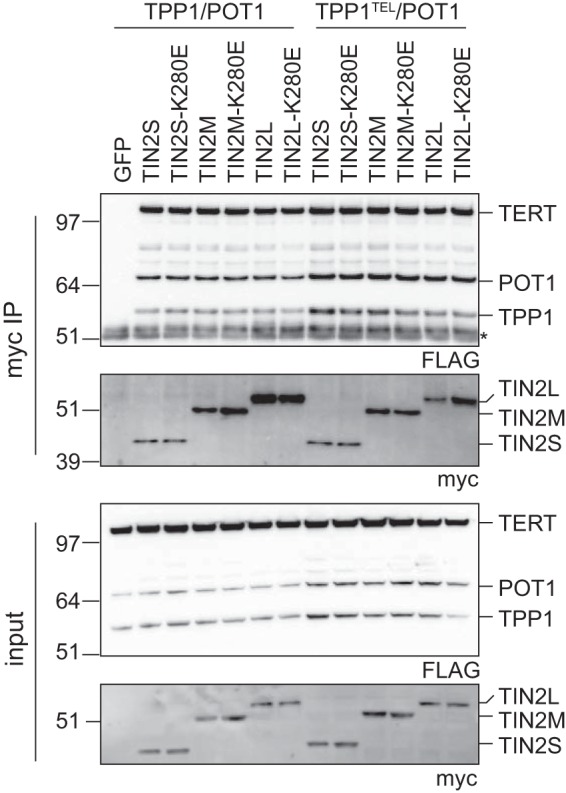
TPP1, POT1, and TERT coimmunoprecipitated with all TIN2 isoforms. myc-tagged TIN2 isoforms were transfected into TPP1/POT1/TERT (left) or TPP1^TEL^/POT1/TERT (right) cell lines. Anti-myc–agarose beads were used to pull down TIN2, and coimmunoprecipitation of FLAG-tagged TERT, TPP1, and POT1was assayed by Western blotting. *, IgG bands; IP, immunoprecipitation assay.

Preliminary experiments showed that the *TINF2* full-length gene stimulated telomerase processivity, and we have demonstrated that each isoform localizes to and functions at telomeres *in vivo*. We next examined whether one of the isoforms might be responsible for the stimulation of telomerase observed with myc-*TINF2*. Each of the three TIN2 isoforms reproducibly coimmunoprecipitated with TPP1/POT1 and TERT in reciprocal pulldown of either myc-TIN2 or FLAG-TPP1/POT1/TERT (Fig. 6 and data not shown). The K280E mutation caused no major change in the coimmunoprecipitation of TPP1/POT1 and TERT in any of the three isoforms (Fig. 6) as had been shown previously for TIN2S (39, 41). Telomerase activity was detected in these coimmunoprecipitations, suggesting that telomerase complexed with TIN2 was active (data not shown). In our hands, TIN2 coimmunoprecipitations showed robust TERT coimmunoprecipitation even in the TPP1^TEL^ mutant, despite previous reports that TEL patch mutations decreased TPP1-TERT interactions by about 40% (20). We conclude that all three isoforms of TIN2 were interacting with TPP1/POT1 in complex with active telomerase and that this complex was not disrupted by the TIN2-K280E patient mutation or the TPP1^TEL^ mutation.

**FIG 7:**
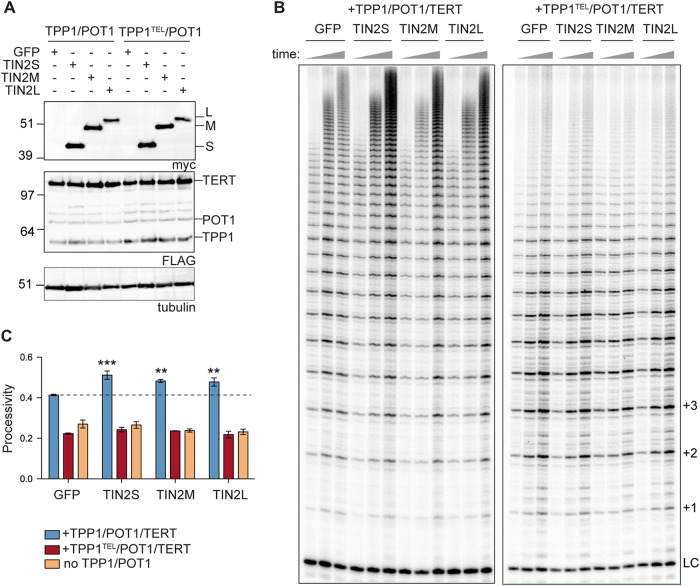
TIN2 stimulates telomerase processivity beyond the TPP1/POT1 stimulation. (A) Western blots of GFP and myc-TIN2 isoform transfections into TPP1/POT1/TERT (left) or TPP1^TEL^/POT1/TERT (right) cell lines. FLAG bands above POT1 are unidentified but may represent TERT degradation products. Tubulin is shown as a loading control. (B) Telomerase assays were stopped at 10, 20, and 40 min (indicated by triangles above the gel). Quantification of the processivity is shown in panel C. LC, loading and purification control; +1, +2, and +3, repeat numbers. (C) Mean processivity values from 3 independent telomerase assays at the 40-min time point using the 15+ processivity method (see Materials and Methods). Orange bars represent a cell line overexpressing TERT/TR but not TPP1/POT1. Data were analyzed with a one-way analysis of variance (ANOVA) and Bonferroni’s multiple-comparison test against the GFP control. *n* = 3 independent transfections per cell line indicated. Error bars represent standard deviations (SD). **, *P* < 0.01; ***, *P* < 0.001.

Using our extract-based telomerase reconstitution system, we transfected TPP1/POT1/TERT cells with each isoform and found reproducible 10% to 20% increase in stimulation of telomerase processivity with each of the three N-terminally tagged isoforms (Fig. 7). TIN2 is important for TPP1/POT1 function in the cell; here, we report evidence of a biochemical function of TIN2 in telomerase processivity stimulation. To determine whether the TIN2 stimulation of processivity works though the TPP1/POT1 heterodimeric telomerase processivity complex, we transfected TIN2 into the TPP1^TEL^/POT1/TERT cells or separately into a TERT-only cell line overexpressing TERT/TR alone. We found no stimulation of telomerase processivity in either of these lysates (Fig. 7C), suggesting that the stimulation was dependent on TPP1/POT1. Our results indicate that TIN2 cooperates with TPP1/POT1 to stimulate telomerase processivity.

Because all three TIN2 isoforms stimulated telomerase processivity in a TPP1/POT1-dependent manner, we tested whether each of patient mutations TIN2-K280E, TIN2-R282S, TIN2-R282H, and TIN2-K280X affects this function of TIN2. In some instances, we found that TIN2 mutants were deficient at stimulating telomerase activity but that this result was variable both in whole-cell lysates and in TIN2 coimmunoprecipitations. Because the *TINF2* mutations are dominant negative *in vivo*, we tried coexpressing wild-type TIN2 with a mutant TIN2, but we were not able to detect a change in processivity stimulation in this *in vitro* setting. Although the patient mutations did not reproducibly affect telomerase processivity in this assay, it is possible that they have important processivity defects *in vivo*.

## DISCUSSION

Our data provide novel biochemical evidence that TIN2 stimulates telomerase processivity in a TPP1/POT1-dependent manner. TIN2 may function by stabilizing the TPP1/POT1 interaction with telomerase, as it relies on TPP1-TERT interaction. This suggests that TIN2 is an integral part of what we refer to as the “TIN2/TPP1/POT1 processivity complex.” This previously unknown role of TIN2 isoforms in telomerase processivity stimulation adds to our understanding of TIN2’s role in telomere length regulation. While all three isoforms were able to stimulate telomerase activity *in vitro*, further work may uncover differences in the regulation of their activities *in vivo*.

### TIN2 cooperates with TPP1/POT1 to stimulate telomerase processivity.

**FIG 8:**
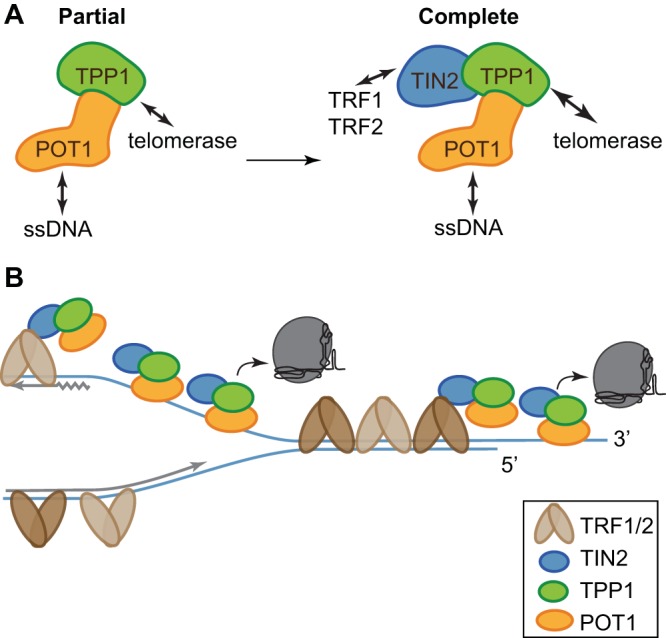
TIN2/TPP1/POT1 forms a stable shelterin subcomplex. (A) TIN2 completes the telomerase processivity complex. TIN2 enhances TPP1/POT1 stimulation of telomerase, forming a heterotrimeric processivity complex. (B) A cartoon proposing a dynamic, heterogeneous distribution of shelterin proteins across the length of human telomeres coordinating telomere length maintenance. TRF1 and TRF2 may direct TIN2/TPP1/POT1 to single-stranded DNA both at the telomere overhang and within the replication fork, aiding its roles in fork progression and telomerase stimulation.

All three TIN2 isoforms formed a stable complex with TPP1/POT1 and TERT, and addition of TIN2 further stimulated telomerase processivity over the level seen with TPP1/POT1 alone. This stimulation of processivity required TPP1 and POT1, as there was no stimulation in cells expressing TPP1 TEL patch mutants or TERT alone (Fig. 7B and C). TIN2 could enhance telomerase processivity by improving the TPP1/POT1 complex stability or its interaction with telomerase, by promoting the telomeric single-stranded DNA (ssDNA) interaction of the complex, or by some combination of the two (Fig. 8A).

Interestingly, the identification of TIN2 as an additional component to an already known processivity complex is reminiscent of recent findings in *Tetrahymena*. The *Tetrahymena* telomerase holoenzyme structure (42) revealed two unknown subunits, Teb2 and Teb3, that interact with the previously defined Teb1-p50 processivity complex. The addition of these proteins to *in vitro* reactions further stimulated telomerase processivity, possibly by stabilizing the completly assembled processive enzyme complex (43). Our results with TIN2 are analogous to this finding in *Tetrahymena*, suggesting that TIN2 binding to TPP1/POT1 stabilizes the complex and thus promotes processivity.

### TIN2/TPP1/POT1 is a functional shelterin subcomplex.

Our data, in combination with previously published work, are consistent with TIN2/TPP1/POT1 forming a shelterin subcomplex. The TPP1-TIN2 interaction is necessary for telomerase recruitment to telomeres (30, 40), even though the TPP1/POT1 heterodimer can bind telomeric ssDNA. Further, experimental data support the idea of different outcomes of the TIN2-TPP1 binding and the TIN2-TRF1 or TIN2-TRF2 binding. First, when TRF1 is removed from telomeres by tankyrase 1 modification, TIN2 and TPP1 remain at telomeres (13). Second, posttranslational depletion of TIN2 by Siah2 ubiquitination removes TPP1 but not TRF1 or TRF2 from telomeres (44). Further evidence in TIN2-floxed mouse cell lines or TIN2 knockdown in HeLa cells showed reduced telomeric TPP1/POT1 localization (29, 30). Similarly, disruption of the TIN2 TRF1-binding motif does not disrupt TRF1, TRF2, or Rap1 localization but prevents TIN2/TPP1/POT1 accumulation at telomeres in mouse cells (28). Deletion of the TPP1-binding region from mouse TIN2 also prevents localization of TPP1/POT1 to telomeres (31). Finally, genetic evidence obtained using CRISPR knockouts in human cells led Kim et al. to conclude that TIN2/TPP1/POT1 is a shelterin subcomplex (45). These findings, together with our work showing a biochemical function of TIN2/TPP1/POT1, further support the conclusion that TIN2, TPP1, and POT1 form a functional shelterin subcomplex.

The TIN2/TPP1/POT1 heterotrimer likely affects both telomerase and replication fork progression. Considering TIN2/TPP1/POT1 to be a telomere-specific ssDNA binding protein (SSB) complex helps explain the defects in telomere replication that have been reported for both POT1 and TPP1 knockdowns and mutants (12, 13, 39, 46, 47). While most diagrams draw TPP1/POT1 bound to the G-strand overhang at telomeres, this telomere-specific SSB complex can also bind the telomeric G strand exposed during DNA replication (Fig. 8B) (29, 48). TPP1 and POT1 have both been reported to facilitate DNA replication through telomeric tracts (49–52). POT1 mutants that cannot bind DNA cause telomere replication fork stalling, fragile telomeres, and ATR activation (52), possibly due to ssDNA exposure at the telomeric replication fork. TIN2 knockdown (29) and mouse mutants (53) also cause an ATR-mediated DNA damage response. Taken together, the data suggest that telomeric TIN2/TPP1/POT1 may participate directly in replication fork progression through the telomere and that perturbation of this function may lead to replication fork collapse and activation of ATR.

### *TINF2* mutations and the role of the TIN2 C terminus in telomere length regulation.

The mutations in *TINF2* in short-telomere syndrome patients mostly cluster in a TIN2 domain of unknown function in exon 6 near the C terminus of TIN2 (25, 26) (Fig. 1A). Genetic evidence strongly supports the idea of a dominant-negative mechanism for the mutant TIN2 proteins, including their autosomal dominant inheritance, stable expression, clustering of disease-associated alleles within the coding sequence, and evidence of selection against the mutant proteins in the hematopoietic lineage *in vivo* (54). Experimental evidence supports the existence of such a dominant-negative mechanism (40), but the molecular nature of this effect is not well understood.

We found that all three TIN2 isoforms form a complex with TPP1/POT1, stimulate telomerase processivity, localize to telomeres, and maintain telomere integrity. Interestingly, these isoforms differ only in their C-terminal domains. TIN2 can be roughly divided into two regions: the shelterin-interacting region in the N terminus and the C-terminal region that includes the patient mutation cluster, the variable C-terminal extensions of TIN2M and TIN2L, and several other interaction sites and modifications (32, 44, 55, 56) (Fig. 1A). The structure is known for much of the shelterin-interacting region, including the N-terminal TRF2/TPP1 binding domain (TIN2_1–202_) (57) and the short TRF1-interacting motif (TIN2_256–276_) (58). There is no structural information, however, regarding the C-terminal region, including both the mutation hot spot and the variable C-terminal extension. Interestingly, the C-terminal extension contains two cell cycle-regulated RSK2 phosphorylation sites (S295 and S333) (56) and a highly conserved region with a CK2 phosphorylation site at S396 (32), while the patient mutation hot spot overlaps a canonical PXVXL HP1γ binding site (55). Additionally, some of the patient mutations, such as K280X, are truncations that generate a short stable protein missing the entire C-terminal region (59). The effects of these binding and phosphorylation events are not yet understood. The TIN2 C terminus may function through binding a novel partner or through a conformational or structural role. Future studies focusing on the C-terminal region of TIN2, especially the conserved region in the C-terminal extension, may reveal novel mechanistic insights that were missed in previous work on TIN2 mutations and telomere shortening.

## MATERIALS AND METHODS

### Expression constructs.

TIN2S cDNA was purchased from Invitrogen (Ultimate ORF Clone IOH80607) in pENTR221. A synthetic gBlock (IDT) containing the downstream TIN2 sequence was used in Gibson assembly to generate TIN2L with silent mutations for shRNA resistance. TIN2M was cloned by reverse transcription-PCR (RT-PCR) of endogenous transcripts. TIN2S, TIN2M, and TIN2L were amplified with primers containing HindIII and NotI restriction sites and an N-terminal myc tag and were cloned into pcDNA5/FRT. *TINF2*, the TIN2 full-length gene inclusive of introns, was cloned into pcDNA5/FRT as described previously (54). Patient mutations were generated by site-directed mutagenesis. The sequences of all constructs and mutants were verified by Sanger sequencing at the Johns Hopkins University (JHU) Synthesis & Sequencing Facility.

p3x-Flag-POT1-cDNA6/Myc-HisC, p3x-Flag-TPP1_87-544_-cDNA6/Myc-HisC, and p3x-Flag-TERT-cDNA6/Myc-HisC were kind gifts from the Cech laboratory (20). We introduced E169A/E171A mutations with site-directed mutagenesis to create TPP1^TEL^. TPP1 or TPP1^TEL^, POT1, and TERT were assembled into a single expression cassette connected by 2A peptides (Fig. 1B). TERT alone was also cloned into pcDNA5. The 2A peptides leave a small tag on the downstream proteins, so TERT was cloned in the last position because it is nonfunctional with C-terminal tags (60–62). Expression cassettes were flanked by BstBI and NotI restriction sites.

### Cell culture.

Cell lines were cultured in the indicated media supplemented with 10% heat-inactivated fetal bovine serum (FBS) (Invitrogen catalog no. 16140071) and 1% penicillin/streptomycin/glutamine (PSG; Invitrogen catalog no. 10378016). HeLa, HeLa TREx FLP-in, 293T, and 293TREx FLP-in cells were cultured in Dulbecco’s modified Eagle’s medium (DMEM; Gibco); hTERT RPE-1 (ATCC CRL-4000) cells were cultured in DMEM–F-12 (Corning); lymphoblastoid cell lines (LCLs) derived from healthy controls (samples were obtained after written informed consent and under approval from Johns Hopkins Medicine Institutional Review Board) were cultured in RPMI medium (Gibco); and K562 cell lines were cultured in Iscove's modified Dulbecco's media (IMDM; Gibco).

For immunofluorescence experiments, the indicated pcDNA5/myc-TIN2 cDNA construct or pcDNA5/GFP was integrated into HeLa FLP-in cells by the use of a FLP-in system (Invitrogen) and hygromycin-resistant clones were pooled for downstream analysis. For TIF experiments, where indicated, cells were also transduced with pGIPZ lentiviruses containing a nontargeting shRNA (Dharmacon catalog no. RHS4346) or shRNA targeting exon 9 of TIN2 (Dharmacon catalog no. V3LHS_401958) at a multiplicity of infection (MOI) of 0.3 and were then selected with puromycin. Cells were analyzed within 2 weeks of transduction to avoid silencing of the shRNA. TIN2S and TIN2M cDNAs have exogenous 3′ untranslated region (3′UTR) sequences and therefore are shRNA resistant; TIN2L was engineered with silent mutations for shRNA resistance in exon 9.

### Telomerase assays.

Telomerase assay cell lines were generated in 293 TREx FLP-in cells (Invitrogen catalog no. R78007) as described previously (63). Briefly, parental cells were transduced with a telomerase RNA (TR) lentivirus, selected, and cloned by limiting dilution. Then, the TPP1/POT1/TERT or TPP1^TEL^/POT1/TERT construct was integrated at a single site in a TR-overexpressing clone using the Flp-in system (Invitrogen). For telomerase assays, 5 × 10^5^ cells of the respective cell lines were plated in each well of a 6-well dish. The next day, 2.5 μg of the indicated TIN2 or GFP construct was transfected with Lipofectamine 2000 (Invitrogen catalog no. 11668019) following the manufacturer’s protocol. After 48 h, cells were lysed in 100 μl 1× CHAPS {3-[(3-cholamidopropyl)-dimethylammonio]-1-propanesulfonate} lysis buffer and clarified by centrifugation. Telomerase assays were performed using 5 μl of clarified cell lysate as described in reference 63. Assays were quantitated in ImageQuantTL (GE Healthcare) using the 15+ method as described previously (20). Statistical analysis was performed in GraphPad Prism.

### Multiple-sequence alignments.

TIN2 sequences from vertebrates with known or predicted TIN2 proteins were obtained from NCBI. The longer isoform was chosen for organisms with multiple reported isoforms. Sequences were uploaded and processed using PRALINE multiple-sequence alignment with the default parameters (64, 65). To make the sequence conservation heat map, the PRALINE output was imported into Microsoft Excel, and the alignment scores (0 to 10) for human TIN2 are indicated on a color-coded scale ranging from white (score of 0 [not conserved]) to navy (score of 8, 9, or 10 [highly conserved]). The sequences used are listed in Table S1 in the supplemental material.

### CRISPR editing.

Guide RNAs were selected using the Zhang Lab CRISPR design tool (http://crispr.mit.edu/). For endogenous tagging of TIN2, the guide sequence (CGCCACCAGGGGCGTAGCCATGG) was cloned into pX459-U6-Chimeric_BB-CBh-hSpCas9-2A-Puro. The repair template was generated by PCR from the cloned myc-*TINF2* construct. A 1-μg volume of Cas9-2A-Puro+TIN2 guide was transfected into 293T cells with 10 molar equivalents of the repair template using XtremeGENE9 (Roche catalog no. 6365787001). The cell population was enriched for edited clones with puromycin, subjected to cloning by limiting dilution, and screened by PCR and restriction digests. Clones with positive results were examined by Western blotting. While we found many edited clones, 293T cells are hypotriploid with an unstable karyotype, and we observed high levels of endogenous Myc expression that interfered with Western blotting for myc-tagged TIN2. These limiting factors make it difficult to further study TIN2 in these knock-in cell lines.

### 3′RACE and PacBio.

The 3′RACE and sequencing assays were performed using samples from five human cell lines (293T, HeLa, RPE-1, K562, and LCL) and two mouse samples (CAST/EiJ mouse embryonic fibroblasts [MEFs] and C57BL/6 liver). All mouse samples were obtained with approval by the Institutional Animal Care and Use Committee at the Johns Hopkins University School of Medicine. We combined 3′RACE with Pacific Biosciences (PacBio) single-molecule, real-time (SMRT) sequencing to cover transcripts from the 5’UTR through the poly(A) tail. First, we isolated mRNA from >10^6^ cells using an RNeasy kit (Qiagen) per manufacturer instructions with QIAshredder spin columns (Qiagen), on-column DNase digestion (Qiagen) to remove any genomic DNA, and an RNA cleanup step. We then reverse transcribed 1.5 μg mRNA with an oligo(dT_20_) primer and an adapter sequence (GACTCGAGTCGACATCG-T_20_) using a SuperScript III first-strand synthesis kit (Qiagen). A 5-μl volume of the resulting cDNA was amplified with Hot Start Phusion polymerase (Thermo) using primers corresponding to the adapter and the 5′-UTR (CGGCGACGTTTAAAGCTGA). A total of 3 to 5 replicate PCRs were combined, purified with a QIAquick PCR purification kit (Qiagen), and submitted to the Johns Hopkins Deep Sequencing & Microarray Core Facility for sequencing. Quality control was performed on a 1:200 dilution of samples using a Bioanalyzer high-sensitivity DNA assay (Agilent). Products were size selected for the anticipated size range of 1 to 3 kb. One SMRT cell was sequenced per sample. Sequencing reads were processed using SMRT Analysis v4.0 software, aligned to chromosome 14 with HISAT2, and assembled into potential transcripts using StringTie (66, 67). StringTie was first run for individual samples using the default settings with the exception that the minimum isoform fraction was set to 0.01 instead of 0.1. To build a gene model for all human reads, StringTie-merge was run with the minimum isoform fraction set to 0.05. HISAT2 and StringTie results were viewed in IGV (68, 69).

### Immunofluorescence.

For TIN2 localization, HeLa FLP-in cells were plated in chamber slides. The following day, the cells were washed with phosphate-buffered saline (PBS) and fixed with 4% paraformaldehyde (PFA) for 20 min. Slides were washed with PBS, treated with 0.5% Triton–PBS for 15 min, washed with PBS, and blocked in 10% goat serum–PBS for 30 min (Sigma). Slides were incubated with a mixture of the two primary antibodies (mouse anti-myc clone 4A6 [Sigma catalog no. 05-724; 1:200] and rabbit anti-TRF2 [Novus Biologicals catalog no. NB110-57130; 1:800]) for 1 h at room temperature, washed with PBS, and incubated with a mixture of the two secondary antibodies (goat anti-mouse IgG1–Alexa Fluor 488 [Invitrogen catalog no. A21121; 1:400] and goat anti-rabbit IgG–Alexa Fluor 555 [Invitrogen catalog no. A21429; 1:400]) for 1 h at room temperature.

For TRF2/53BP1 colocalization analysis, cells were seeded in chamber slides and, 2 days later, samples were processed for immunofluorescence staining. Before fixation was performed, cells were subjected to *in situ* fractionation as described previously (70). After several washes with cold PBS were performed, the cells were ﬁxed in 3% paraformaldehyde–2% sucrose–PBS for 10 min at room temperature, washed in PBS, and permeabilized in cold 0.3% Triton–PBS for 8 min. After three PBS washes, the slides were blocked in 3% goat serum–1 mg/ml bovine serum albumin (BSA)–PBS for 30 min at room temperature, incubated with primary antibodies (rabbit anti-Trf2 [NB110-57130; Novus Biologicals; 1:800] and mouse anti-53BP1 [NBP2-25028; Novus Biologicals; 1:400]) diluted in blocking solution for 1 h at room temperature. After three PBS washes, the slides were incubated with secondary antibodies (goat anti-rabbit IgG–Alexa Fluor 555 [A21429; Invitrogen; 1:400] and goat anti-mouse IgG–Alexa Fluor 647 [A21235; Invitrogen; 1:400]) for 45 min at room temperature.

All stained slides were washed three times with PBS and coverslips were mounted with Vectashield containing DAPI (4′,6-diamidino-2-phenylindole) after the secondary antibody incubation. Images were acquired on a Nikon Eclipse NI-E microscope using a PlanApo 60× objective and NIS-Elements software.

### TIF analysis.

Colocalization analysis was performed using Nikon NIS-Elements software. Nuclei were identified using thresholding as regions of interest and were excluded if they were not completely in the field of view. Thresholding was performed on the Trf2 and 53BP1 images separately to identify individual foci. Nuclei were excluded if they contained multiple foci combined into one (Trf2 and 53BP1) or if they contained ≤5 foci (Trf2 only). An intersection layer was created from the Trf2 and 53BP1 layers to identify colocalized foci, and the data for each ROI layer were exported to a data table. Descriptive statistics were generated, and data were analyzed by Kruskal-Wallis nonparametric analysis of variance with multiple-comparison corrections performed using GraphPad Prism 8. Data were visualized as a box plot with whiskers covering all values for each data set. Representative nuclei were selected in Nikon NIS-Elements software, and image contrast was enhanced for visual presentation postanalysis.

### qRT-PCR.

RNA was purified from >10^6^ cells using a Qiagen RNeasy kit, and 2 μg of mRNA was reverse transcribed using a SuperScript III first-strand synthesis kit (Invitrogen) with oligo(dT_20_) primers. Reverse transcription-quantitative PCR (qRT-PCR) was performed on a Bio-Rad CFX96 thermocycler with 1× IQ SYBR green Supermix (Bio-Rad). TIN2 (primers: GTCAGAGGCTCCTGTGGATT and CAGTGCTTTCTCCAGCTGAC) was measured against the ARF3 housekeeping gene (primers: TCACCACCATCCCTACCATT and AGGTGGCCTGAATGTACCAG) (34). Data were analyzed using the threshold cycle (2^−ΔΔ^*^CT^*) method of relative quantification.

### Western blotting.

Cells were lysed on ice in CHAPS lysis buffer (10 mM Tris-HCl, 1 mM MgCl_2_, 1 mM EGTA [pH 8.0], 0.1 mM benzamidine, 5 mM β-mercaptoethanol [BME], 0.5% CHAPS, 10% glycerol [pH 7.5]) and clarified by centrifugation. Samples were denatured with 1× NuPAGE LDS sample buffer Invitrogen catalog no. NP0008) mixed with 50 mM dithiothreitol (DTT), heated at 65°C for 10 min, and separated on a 4% to 12% Bis-Tris gel (NuPAGE catalog no. NP0323) in 1× MOPS (morpholinepropanesulfonic acid) buffer (Invitrogen catalog no. NP0001) with 3 μl of SeeBlue Plus2 (Thermo catalog no. LC5925) with a prestained ladder to estimate molecular weight. Proteins were transferred to polyvinylidene difluoride (PVDF), blocked in 1× TBS–0.1% Tween 20 (TBST)–5% milk (Bio-Rad catalog no. 170-6404), probed with the indicated antibodies, and developed by chemiluminescence using an ImageQuant LAS4000 imager (GE Healthcare). Primary antibodies and concentrations were as follows: mouse anti-myc 4A6 (Millipore catalog no. 05-24; 1:2,000), mouse anti-FLAG M2 (Sigma catalog no. F1804; 1:5,000), and rabbit antitubulin (Abcam catalog no. ab6046; 1:5,000). Secondary antibodies were anti-mouse IgG or anti-rabbit IgG conjugated to horseradish peroxidase (HRP; Cell Signaling; 1:10,000).

### Coimmunoprecipitation.

Immunoprecipitations were carried out using either anti-c-*myc*–agarose (Pierce 20168) or anti-FLAG M2 affinity gel (Sigma A2220). A 20-μl volume of bead slurry per reaction was washed with PBS and equilibrated in CHAPS buffer before addition of 45 μl of lysate. Samples were incubated in an end-over-end mixer at 4°C for 2 h. Beads were pelleted, washed 4 times with 300 μl 1× CHAPS buffer, and resuspended in 2× LDS loading dye for Western blot analysis.

## Supplementary Material

Supplemental file 1
