# Supplementary material for: TIN2 Functions with TPP1/POT1 To Stimulate Telomerase Processivity
Source: Mol Cell Biol. 2019 Oct 11;39(21):e00593-18. doi: 10.1128/MCB.00593-18 (PMC6791651; doi:10.1128/MCB.00593-18)
Supplement: Supplemental file 1 [file MCB.00593-18-s0001.pdf]

Supplementary Table 1. Sequences used for TIN2 multiple sequence alignment

Species	Accession Number	Species	Accession Number
Homo sapiens	NP_001092744.1	*Canis lupus*	XP_005623322.1
Pan troglodytes	NP_001233535.1	*Bos taurus*	NP_001091583.1
Pan paniscus	XP_003809126.1	*Equus caballus*	XP_005603304.1
Pongo abelii	NP_001124653.1	*Mustela putorius furo*	XP_004755289.1
Chlorocebus sabaeus	XP_007984509.1	*Felis catus*	XP_006932836.1
Macaca fascicularis	XP_005561020.1	*Mus musculus*	NP_663751.2
Macaca mulatta	XP_014998924.1	*Mus spretus*	AAN77121.1
Callithrix jacchus	XP_002807259.1	*Rattus norvegicus*	NP_001006963.1
Cricetulus griseus	EGW11547.1	*Myotis brandtii*	EPQ11041.1
Ictidomys tridecemlineatus	XP_005338777.1	*Loxodonta africana*	XP_003421012.2
Saimiri boliviensis	XP_010333305.1	*Sarcophilus harrisii*	XP_012400471.1
Heterocephalus glaber	EHB03391.1	*Alligator sinensis*	XP_006036607.1
Oryctolagus cuniculus	XP_008267657.1	*Xenopus laevis*	AAI26059.1
Ochotona princeps	XP_012782354.1	*Xenopus tropicalis*	ACC76751.1
Sus scrofa	XP_001927960.1	*Danio rerio*	XP_005172357.1
Ovis aries	XP_012036332.1	*Monodelphis domestica*	XP_007479934.1
Tursiops truncates	XP_004311674.1	*Anolis carolinensis*	XP_008117770.
Vicugna pacos	XP_006217349.1		
